# Apresentação Rara de Tumor de Saco Vitelino com Envolvimento Cardíaco: Características Detectadas pela Ressonância Magnética

**DOI:** 10.36660/abc.20210335

**Published:** 2022-07-07

**Authors:** Cristhian Espinoza Romero, Williams Roberto Lata Guacho, Kevin Rafael de Paula, Robert Paladines Jimenez, Eduardo Kaiser Ururahy Nunes Fonseca

**Affiliations:** 1 Universidade de São Paulo Instituto do Coração São Paulo SP Brasil Universidade de São Paulo – Instituto do Coração, São Paulo, SP – Brasil

**Keywords:** Tumores Cardíacos, Insuficiência Cardíaca, Neoplasias Embrionárias de Células Germinativas, Diagnóstico por Imagem, Ecocardiografia Transtorácica/métodos, Espectroscopia de Ressonância Magnética/métodos, Tratamento Farmacológico

## Introdução

Os tumores cardíacos primários são extremadamente raros, com uma incidência variável entre 0,0017 e 0,28%, dentro desses encontra-se o tumor de células germinativas de tipo saco vitelino (TSV) de caráter maligno.^1^

Embora o ecocardiograma transtorácico (ETT) muitas vezes seja a primeira linha na avaliação de tumores cardíacos, atualmente, em virtude de sua boa resolução espacial e a caracterização tecidular, a ressonância magnética cardíaca (RMC) é a técnica de eleição na avaliação desses tumores.^2,3^ O TSV de localização intracardíaca é rara, sendo poucos os casos relatados.^4–7^

## Relato de caso

**Figura 1:**
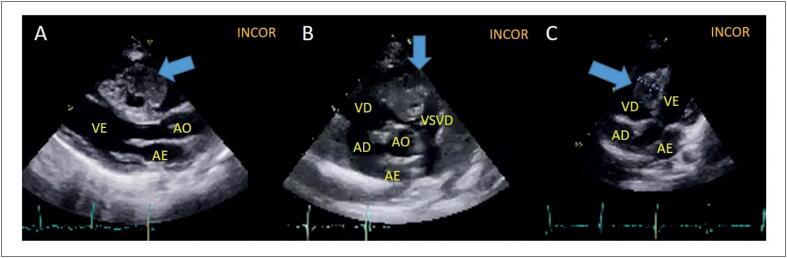
Ecocardiograma transtorácico. (A) Plano longitudinal 3 câmaras em diástole mostrando massa heterogênea no VD (seta). (B) Eixo curto com sinais de obstrução da VSVD (seta). (C) Plano coronal 4 câmaras imagem lobulada com projeção para o VD (seta). VE: ventrículo esquerdo; VD: ventrículo direito; AE: átrio esquerdo; Ao: aorta; AD: átrio direito; VSVD: via de saída do ventrículo direito.

Uma paciente do sexo feminino, de 1 ano de idade, apresentou-se com episódios de cianose ao chorar. Ao exame físico apresentava frequência cardíaca de 132 bpm, sopro sistólico 2+/6+, desdobramento fixo de segunda bulha cardíaca, perfusão adequada, com pulsos amplos. Pelos sinais de insuficiência cardíaca, foi realizado um ETT evidenciando uma massa heterogênea e multilobulada no ventrículo direito (VD), junto ao septo interventricular, com área estimada de 7,8cm², algumas áreas císticas e sinais de calcificação com sinais de obstrução na via de saída do ventrículo direito (VSVD) (Figura 1).

**Figura 2:**
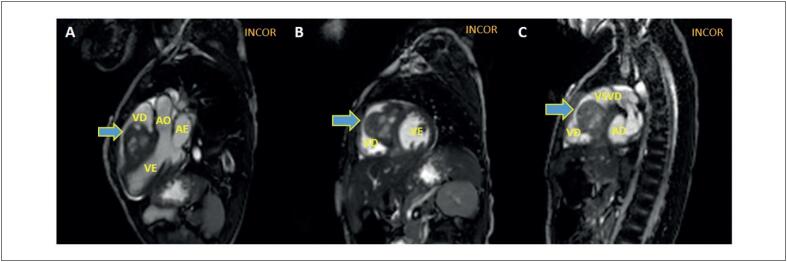
Ressonância magnética cardíaca com sequência de pulso “steady-state free precession”. (A) Plano longitudinal 3 câmaras em sístole mostrando massa expansiva localizada no septo intraventricular (seta). (B) Plano axial eixo curto exibe massa com extensão para VD (seta). (C) Plano axial eixo curto, observa-se obstrução do tumor na VSVD (seta). VE: ventrículo esquerdo; VD: ventrículo direito; AE: átrio esquerdo; Ao: aorta; AD: átrio direito; VSVD: via de saída do ventrículo direito.

Foi feita RMC (Figuras 2 e 3) que demostrou formação expansiva com ampla base de inserção no septo interventricular, sem plano de clivagem com o miocárdio adjacente, de contornos lobulados, estendendo-se para a cavidade do VD, medindo aproximadamente 38 x 35 x 43 mm. Essa lesão apresentava áreas císticas de permeio, exibindo baixo sinal heterogêneo em T1 e discreto alto sinal igualmente heterogêneo em T2, além de impregnação heterogênea pelo gadolínio na sequência de realce tardio (RTG) e captação do contraste na sequência de perfusão. Foi realizada biopsia de lesão pulmonar descrita como neoplasia maligna epiteloide com extensa necrose, com índice mitótico de 10 mitoses x campo e imuno-histoquímico positivo para SALL4, alfa-fetoproteína e PLAP nas células de interesse, sendo consistente para neoplasia de células germinativas, compatível com tumor de saco vitelino.

**Figura 3:**
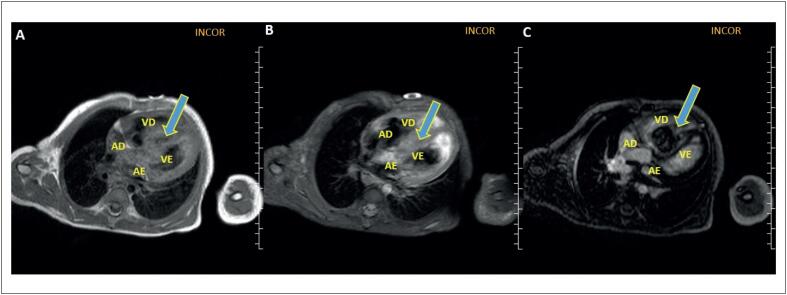
Ressonância magnética cardíaca. Características teciduais (A) Sequência sem contraste FSE com saturação de gordura, plano coronal 4 câmaras, mostra hiposinal heterogêneo do septo (seta). (B) Sequência sem contraste FSE ponderada em T2 com tripla inversão-recuperação, plano axial 4 câmaras, mostra mínimo aumento de sinal heterogéneo do septo (seta). (C) Sequência de realce tardio, plano coronal 4 câmaras presença de realce tardio heterogêneo do septo (seta). VE: ventrículo esquerdo; VD: ventrículo direito; AE: átrio esquerdo; AD: átrio direito.

A paciente foi submetida a quimioterapia com cisplatino, porém em controles evolutivos não houve alterações significativas dos achados no ETT. Atualmente, programa-se abordagem cirúrgica devido à refratariedade à quimioterapia.

## Discussão

As características dos tumores cardíacos malignos, dentro os quais estão os germinativos, tem sido estudada em algumas revisões. Considera-se a RMC o método de escolha para sua avaliação, visto que tem uma alta acurácia em discriminar lesões benignas de malignas, por avaliar a localização, tamanho e contornos da lesão. Além disso, a RMC tem um valor diagnóstico significativo para as características do sinal dos componentes do tecido dentro dos tumores, incluindo calcificação, gordura, fibrose, hemorragia, e mudanças císticas.^8^ Dos tumores de células germinativas as principais características visualizadas por RMC são o realce tardio com gadolínio heterogêneo e na cine-ressonância e nas sequencias ponderadas em T1 e T2, também uma intensidade heterogênea.^8^

Das principais características que sugerem malignidade temos as dimensões >5cm, contornos irregulares, lesões múltiplas, envolvimento pleural ou pericárdico, invasão direta dos planos dos tecidos, localização no coração direito, e características tecidulares tais como a heterogeneidade de sinal nas sequências ponderadas em T1 e T2 e presença de realce com contraste na primeira passagem sugerindo vascularização da lesão.^9,10^

Assim, destacamos a grande utilidade da RMC, neste caso, como auxiliar no diagnóstico e suspeita de um tumor de etiologia maligna, por meio de algumas das características já descritas, como a localização no VD, contornos mais irregulares, sinais heterogêneos nas sequências T1 e T2 e RTG, além de captação de contraste na sequência de perfusão, que foram descritas em nosso paciente.

O tratamento padrão dos tumores primários não seminomatosos, tal qual o TSV, é uma combinação de quimioterapia sistêmica neoadjuvante com bleomicina ou cisplatina, com tentativa de ressecção cirúrgica.^11^

Foi relatado um caso muito infrequente de tumor cardíaco primário de saco vitelino com características malignas confirmado por biopsia sem adequada resposta à quimioterapia. A RMC da paciente apresentou algumas das características que acrescentaram a possibilidade de malignidade como o tamanho e o RTG heterogéneo. Atualmente as técnicas de imagem como a RMC são de grande ajuda, e em alguns casos são os métodos de escolha para um adequado diagnóstico.
