# Supplementary material for: Apresentação Rara de Tumor de Saco Vitelino com Envolvimento Cardíaco: Características Detectadas pela Ressonância Magnética
Source: Arq Bras Cardiol. 2022 Jul 7;119(1):136–8. [Article in Portuguese] doi: 10.36660/abc.20210335 (PMC9352127; doi:10.36660/abc.20210335)
Supplement: Translation

# Translation: Rare Presentation of Yolk Sac Tumor with Cardiac Involvement: Characteristics Detected by MRI

**Authors:** Cristhian Espinoza Romero, Williams Roberto Lata Guacho, Kevin Rafael de Paula, Robert Paladines Jimenez, Eduardo Kaiser Ururahy Nunes Fonseca

**Affiliations:** 1 São Paulo SP Brazil Universidade de São Paulo – Instituto do Coração, São Paulo, SP – Brazil

**Keywords:** Heart Neoplasms, Heart Failure, Neoplasms, Germ Cell and Embryonal, Diagnostic, Imaging, Echocardiography, Transthoracic/methods, Magnetic Resonance, Spectroscopy/methods, Drug Therapy

## Introduction

Primary cardiac tumors are extremely rare, with their incidence varying between 0.0017 and 0.28%, among which is the malignant germ cell yolk sac tumor (YST).^1^

Although transthoracic echocardiography (TTE) is often the first line in the evaluation of cardiac tumors, currently, due to its good spatial resolution and tissue characterization, cardiac magnetic resonance (CMR) imaging is the technique of choice for the evaluation of these tumors.^2,3^ The intracardiac YST is a rare neoplasm, with few reported cases.^4–7^

## Case report

A one-year-old female patient presented with episodes of cyanosis when crying. On physical examination, she had a heart rate of 132 bpm, a 2+/6+ systolic murmur, fixed splitting of the second heart sound, adequate perfusion, with wide pulses. Due to the signs of heart failure, a TTE was performed, which showed a heterogeneous and multilobulated mass in the right ventricle (RV), next to the interventricular septum, with an estimated area of 7.8 cm², some cystic areas and signs of calcification, with signs of obstruction in the right ventricle outflow tract (RVOT) (Figure 1).

**Figure 1:** Transthoracic echocardiogram. (A) Longitudinal 3-chamber view in diastole showing heterogeneous mass in the RV (arrow). (B) Short axis with signs of RVOT obstruction (arrow). (C) Coronal 4-chamber lobulated image with projection to the RV (arrow). LV: left ventricle; RV: right ventricle; LA: left atrium; AO: aorta; RA: right atrium; RVOT: right ventricular outflow tract.

A CMR (Figures 2 and 3) was performed, which showed an expansive formation with a wide insertion base in the interventricular septum, showing no cleavage plane with the adjacent myocardium, with lobulated contours, extending into the RV cavity, measuring approximately 38 x 35 x 43 mm. This lesion had intermingled cystic areas, exhibiting low heterogeneous signal on T1 and a slightly high, equally heterogeneous signal on T2, in addition to heterogeneous gadolinium uptake in the late gadolinium enhancement (LGE) sequence, and contrast uptake in the perfusion sequence. The biopsy of a pulmonary lesion described as an epithelioid malignant neoplasm with extensive necrosis was performed, with a mitotic index of 10 mitoses x field and positive immunohistochemistry for SALL4, alpha-fetoprotein and PLAP in the cells of interest, being consistent with a germ cell neoplasm, compatible with a yolk sac tumor.

**Figure 2:** Cardiac magnetic resonance with steady-state free precession pulse sequence. (A) Longitudinal 3-chamber view in systole showing an expansive mass located in the intraventricular septum (arrow). (B) Short axis axial view showing mass with extension to the RV (arrow). (C) Short-axis axial view showing tumor obstruction in the RVOT (arrow). LV: left ventricle; RV: right ventricle; LA: left atrium; AO: aorta; RA: right atrium; RVOT: right ventricular outflow tract.

**Figure 3:** Cardiac magnetic resonance imaging. Tissue characteristics (A) FSE sequence without contrast, with fat saturation, 4-chamber coronal view, showing heterogeneous hyposignal in the septum (arrow). (B) T2-weighted FSE sequence without contrast, with triple inversion-recovery, 4-chamber axial view, showing a minimal increase in heterogeneous signal in the septum (arrow). (C) Late enhancement sequence, 4-chamber coronal view, presence of heterogeneous delayed enhancement in the septum (arrow). LV: left ventricle; RV: right ventricle; LA: left atrium; RA: right atrium.

The patient was submitted to chemotherapy with cisplatin, but the control exams to assess disease evolution showed no significant changes in the TTE findings. Currently, a surgical approach is scheduled due to refractoriness to the chemotherapy.

## Discussion

The characteristics of malignant cardiac tumors, which include the germ cell ones, have been studied in some reviews. CMR imaging is considered the method of choice for their evaluation, since it shows high accuracy in discriminating benign from malignant lesions, assesses the location, size and contours of the lesion. Moreover, the CMR has a significant diagnostic value for the signal characteristics of tissue components inside the tumors, including calcification, fat, fibrosis, hemorrhage, and cystic changes.^8^ Of the germ cell tumors, the main characteristics visualized by CMR are the delayed heterogeneous gadolinium enhancement, and on cine-resonance and T1- and T2-weighted sequences, also a heterogeneous intensity.^8^

Among the main features that suggest malignancy are tumor size >5cm, irregular contours, multiple lesions, pleural or pericardial involvement, direct invasion of tissue planes, right heart location, and tissue characteristics such as signal heterogeneity on T1- and T2-weighted sequences and presence of contrast enhancement in the first pass, suggesting lesion vascularization.^9,10^

Therefore, we highlight the great usefulness of CMR, in this case, as an aid in the diagnosis and suspicion of a tumor of malignant etiology through some of the previously described characteristics, such as location in the RV, more irregular contours, heterogeneous signals in the T1 and T2 and LGE, in addition to contrast uptake in the perfusion sequence, which were described in our patient.

The standard treatment of primary nonseminomatous tumors, such as the YST, is a combination of neoadjuvant systemic chemotherapy with bleomycin or cisplatin, together with attempted surgical resection.^11^

This case report describes a very rare case of primary cardiac yolk sac tumor with malignant features confirmed by biopsy, which did not show an adequate response to chemotherapy. The patient’s CMR showed some of the characteristics that added to the possibility of malignancy, such as the size and heterogeneous LGE. Currently, imaging techniques such as CMR are very useful and, in some cases, they constitute the methods of choice to attain an adequate diagnosis.
